# Artificial Structural Color Pixels: A Review

**DOI:** 10.3390/ma10080944

**Published:** 2017-08-14

**Authors:** Yuqian Zhao, Yong Zhao, Sheng Hu, Jiangtao Lv, Yu Ying, Gediminas Gervinskas, Guangyuan Si

**Affiliations:** 1 College of Information Science and Engineering, Northeastern University, Shenyang 110004, China; zhaoyong@ise.neu.edu.cn (Y.Z.); husheng2008@hotmail.com (S.H.); youjing56789@gmail.com (J.L.); 2 College of Information & Control Engineering, Shenyang Jianzhu University, Shenyang 110168, China; yingyu@sjzu.edu.cn; 3 Melbourne Centre for Nanofabrication, Clayton, Victoria 3168, Australia; gediminas.gervinskas@monash.edu

**Keywords:** color filters, photonic crystals, plasmonics, tunable devices

## Abstract

Inspired by natural photonic structures (Morpho butterfly, for instance), researchers have demonstrated varying artificial color display devices using different designs. Photonic-crystal/plasmonic color filters have drawn increasing attention most recently. In this review article, we show the developing trend of artificial structural color pixels from photonic crystals to plasmonic nanostructures. Such devices normally utilize the distinctive optical features of photonic/plasmon resonance, resulting in high compatibility with current display and imaging technologies. Moreover, dynamical color filtering devices are highly desirable because tunable optical components are critical for developing new optical platforms which can be integrated or combined with other existing imaging and display techniques. Thus, extensive promising potential applications have been triggered and enabled including more abundant functionalities in integrated optics and nanophotonics.

## 1. Introduction

Rather than using colorant-based pigmentation, structural color filtering devices [1,2,3,4,5,6,7,8] normally take advantage of geometry-dependent spectra resonance due to their particular capability [9,10,11,12,13,14,15,16,17,18] of manipulating light at the nanoscale. Particularly, color generation based on geometry control [19,20,21,22,23,24,25,26] rests with tuning of resonance in visible frequencies via manipulating structural parameters [27,28,29,30,31]. For existent digital imaging technologies, primary color components (e.g., red, green, and blue) can be precisely balanced in each pixel, thereby producing various colors. With the fast development of nanofabrication and characterization technologies [32,33,34,35,36,37,38], photonic/plasmonic crystals and related nanodevices [39,40,41,42,43,44,45] have already generated significant interest in recent years. Using diverse nanofabrication techniques, both transmissive and reflective structural color filters have been demonstrated [46,47,48,49]. Filter-free image sensor pixels [50] have also been experimentally studied. In addition, the effect of relative nanohole position on color purity [51] has been investigated. The working principles of artificial structural color devices are using geometrical resonances to manipulate light in visible frequencies and further engineer surface waves at the nanoscale [52,53,54,55,56,57,58,59,60,61,62,63,64], which is different from traditional optical devices such as fiber Bragg gratings or other constructive interference optical components [65,66,67,68].

Furthermore, investigations on magnetophotonic composites and relevant devices [69,70,71,72,73] have drawn significant attention recently. Using a synthesis method which combines a sol-gel route followed by a reduction step, three dimensional network magnetophotonic crystals can be fabricated on Morpho butterfly wing templates [74]. Moreover, magnetophotonic heterostructure based dual-channel sensors [75] and stop band reconfiguration in one-dimensional magnetophotonic crystals [76] have been studied, respectively. Parvini and coworkers proposed the concept of defective magneto-optical photonic crystal based color filters [77] and designed incident angle variant devices which can cover the whole visible band. Most recently, Guay et al. demonstrated laser-induced plasmonic colors on metals and this bottom-up approach is suitable for high-throughput industrial applications [78]. Although numerous practical applications have been triggered by photonic/plasmonic optical components due to their particular ability of manipulating light at nanoscales, the recent growing interest in mobile and electronic-book devices still has challenging demands for new display technologies since the advancement of display technology has played a critical role in improving daily life quality. Such devices can find wide applications in optical filters and metasurfaces for controlling wavefront, enabling high compactness and resolution beyond the diffraction limit with wide tunability and highly stable device performance. Many other applications (metasurface holograms, for instance) can be triggered using similar principles since localized surface plasmon resonance (LSPR) is intrinsically dependent on the incident light polarization state. Among all these applications, color filtering components are extremely important due to their significant potential for imaging and display technologies. Plasmonic based gratings in metallic surfaces can avoid involving diffraction effects, enabling independency of the angle of incidence. By avoiding grating coupling for mode excitation, one can realize angle robust optical devices [6] which make use of metal-insulator-metal Fabry-Pérot cavity modes to minimize the angle dependence with small pixel sizes. Displays with low power consumption and full-color emission under a broad range of ambient lighting conditions are highly desired. In this review, we summarize the recent progress of structural color filtering devices which are critical and essential for nanophotonics and display technologies. Different fabrication technologies and working principles of both photonic crystals and plasmonic-assisted filtering devices which can cover the entire visible band are reviewed and further elaborated here.

## 2. Photonic Crystal Color Pixels

### 2.1. Structural Colors Observed in Nature

**Figure 1:**
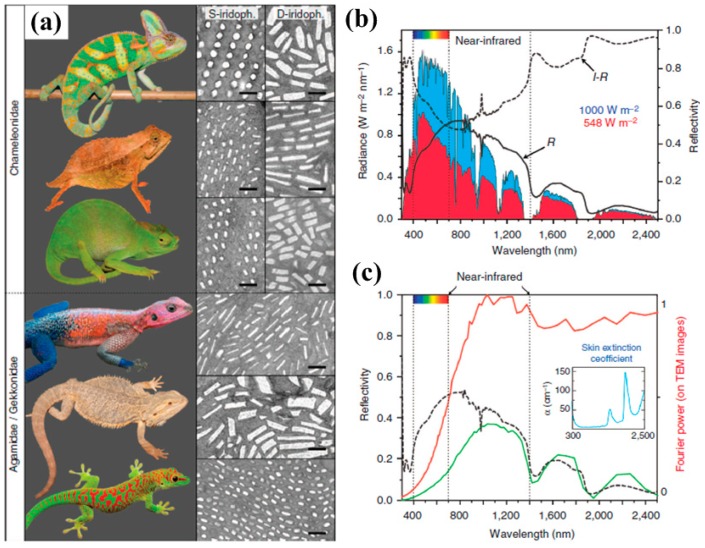
(**a**) Chamaeleonidae (top to bottom: Chamaeleo calyptratus, Rhampholeon spectrum and Kinyongia matschiei) exhibit two superposed layers of (S- and D-) iridophores, whereas agamids (the sister group to chameleons) and gekkonids have a single-type iridophore layer (top to bottom: Agama mwanzae, Pogona vitticeps, and Phelsuma grandis). Scale bars, 500 nm; (**b**) Reflectivity (R) of a panther chameleon white skin sample and solar radiation spectrum (blue curve) at sea level; (**c**) Fourier power spectrum and reflectivity of a panther chameleon red skin sample. Inset, skin extinction coefficient as a function of wavelength. All figures are adapted from reference [80].

In general, the working principle of dyes or pigments is based on light-matter interaction which in fact is that they can absorb light of only certain wavelengths and reflect the remaining frequency bands of the spectrum, leading to resonance peaks in either transmission or reflection. People have long benefited from nature’s capability of creating coloration. The famous Morpho butterfly wings [79] were one of the first examples studied by researchers decades ago and they normally shine a dazzling blue color. Another well-known example is chameleon. Some chameleons can also alter colors rapidly according to the change of background or environment. Recent research [80] has found that chameleons can shift color by dynamically adjusting the lattice of guanine nanocrystals within a superficial thick layer of dermal iridophores. As shown in Figure 1a, chamaeleonidae (top panel) show two superposed layers of iridophores but their sister group, agamidae/gekkonidae, only exhibit a single-type of iridophore layer. Interestingly, the upper layer (superficial (S-) iridophores) is composed of small close-packed guanine crystals while the deeper iridophores (deep (D-) iridophores) comprise larger disorganized guanine crystals which mainly reflect light in near-infrared frequencies. Moreover, the S-iridophores are only completely developed in the skin of adult males with guanine crystal diameter ≈130–140 nm. On the other hand, the D-iridophores can be found in all panther chameleons which can abate the absorption of sunlight. Figure 1b plots the reflectivity of a panther chameleon white skin sample illuminated by a solar radiation spectrum and the measurements reveal that relatively high intensity can be observed in the near-infrared wavelength range. If we multiply the sun radiance (blue curve) by 1–R, to yield the amount of light transmitted by the dermis (red curve), one can see that the radiation energy in that frequency range is screened in panther chameleons by reflection on the dermis. In order to further test whether this infrared reflectivity is probably due to coherent scattering on guanine crystals in D-iridophores, the computed Fourier power spectrum as an estimate of the spectral shape (red curve in Figure 1c) shows the light backscattered by deep iridophores, indicating that the D-iridophore layer can act as a broad-band reflector in the near infrared region. It is also known that some chameleons’ territories are in open country and normally they are exposed to sunlight directly. This special design of dual-layer iridophores enables the combination of impactful camouflage and dazzling display.

### 2.2. Reflective Photonic Crystal Color Filter Development

**Figure 2:**
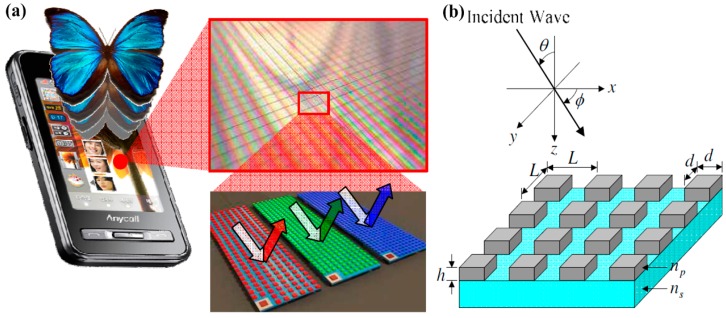
(**a**) Schematic diagram showing the reflective photonic crystal color filters and (**b**) the sketch of its geometry. All figures are adapted from reference [81].

Inspired by distinctive Morpho butterfly wings, reflective photonic crystal color filters consisting of nanogratings were proposed and experimentally fabricated [81]. As shown in Figure 2, the two-dimensional (2D) nanograting array is composed of silicon (refractive index = *n_p_*) on top of a glass substrate (refractive index = *n_s_*), which can function as a planar waveguide. Periodicity, width, and height are labeled as *L*, *d* and *h*, respectively. Generally speaking, these 2D nanogratings can work as filters under phase-matching conditions. Diffracted waves can be generated and the reflected energy can be dramatically strengthened within certain frequency ranges. These 2D color filters exhibit unique optical properties compared with 1D nanogratings which have strong angle dependency on incident light. Theoretically, one can avoid relying on grating coupling for resonance mode excitation to minimize angle sensitivity. Different from grating coupling, plasmon-assisted resonators and nano-antennae normally need a large density for effectively scattering light to either viewers’ eyes or photodetectors in the visible range. The angle dependence for plasmonic crystals is directly related to the surface plasmon polariton excitation via grating coupling which limits the practical applications due to relatively low coupling efficiencies since it is inherently angle-dependent because of momentum matching conditions. Overcoming this angle-dependent spectrum response will allow these structural filters to be integrated into practical applications such as high resolution visual displays, miniature hyperspectral imaging, and high sensitivity sensing devices.

Note that these 2D photonic crystal filters utilized the coupling between the incident light and the resonant modes (guided-mode resonance) to generate individual colors. Such filters show high reflectance (>70%) and angular tolerance and they can suppress the incident-angle dependency and enhance the chromatic properties (smaller spectrum bandwidth) compared with traditional 1D color filters, leading to useful applications for display techniques. The geometry can also be further optimized to make the optical effect close to being independent of the tilt angle and therefore enable wide use in ambient light conditions, paving the way to more practical applications in displays. The pioneering experiments on angle robust optical devices were performed by Wu and coworkers [6] who utilized metal-insulator-metal Fabry–Pérot cavity modes to avoid relying on grating coupling for mode excitation, enabling angle insensitive color filters up to ±80° with the pixel size as small as λ/2.

**Figure 3:**
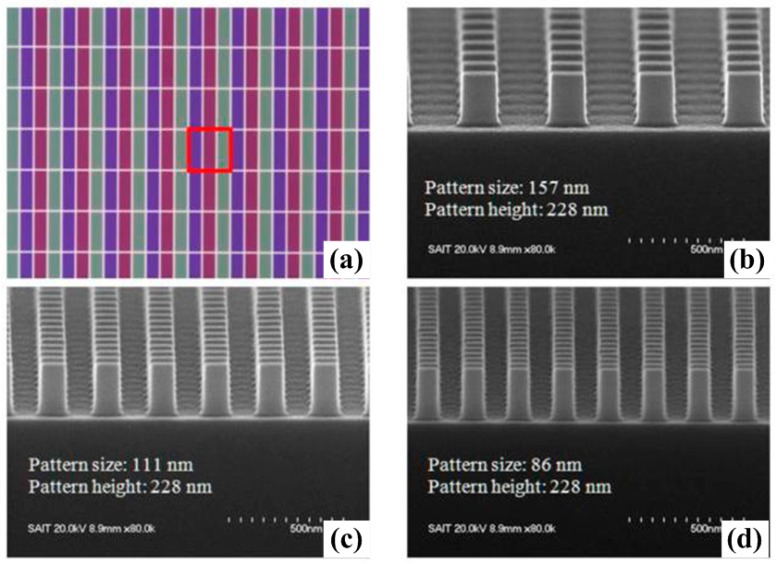
(**a**) Optical microscope image of photonic crystal array. Scanning electron microscopy (SEM) images of the 2D pillar pattern for (**b**) red; (**c**) green; and (**d**) blue color filters. All figures are adapted from reference [82].

Furthermore, the reflectivity for s- and p-polarization is different. For s-polarization, it does not change greatly in the Brillouin zone, while for p-polarization it lessens through the range. Thanks to the fast development of nanotechnology, color filters can be fabricated by using electron-beam lithography (EBL) and nanoimprint lithography (NIL) followed by inductively coupled plasma reactive ion etching (ICP-RIE) to transfer patterns from resists to silicon substrates. However, one should note that the crystallinity of the fabricated silicon color filters may affect the device performance significantly. For instance, the maximum reflectance is reduced tremendously for amorphous silicon due to large internal absorption. Therefore, the reflectance spectrum may vary due to different silicon crystallinity properties and various refractive indices. Using NIL and multi-scan excimer laser annealing, 2D photonic crystal color filters were experimentally demonstrated by Cho and coworkers [82]. This concept utilizes 2D subwavelength photonic crystals for reflective displays. Figure 3a shows the optical microscope image of RGB colors generated. Scanning electron microscopy (SEM) micrographs show uniform cross sections of the nanogratings fabricated. Pillars 228-nm-high have been achieved after dry etching for pattern transfer. Different color outputs can be simply realized by adjusting the geometric parameters, resulting in a highly compositive platform for imaging and display nanophotonics. More importantly, very small (±2%) critical dimension tolerance has been realized with less than 5 nm edge roughness. The pattern pitch for each color filter did not change during the etching process. Note that one can easily actualize tunable color filtering devices by using crystalline colloidal arrays with an external electric field. Optical properties like bandwidth and bandgap position can be effectively modulated by controlling the refractive index of the particle, enabling full color adjustment and novel reflective display devices. Alternatively, one can utilize flexible polymer to bring about tunable optical effects [83,84,85] through mechanical force with nano-/micro-electromechanical system actuators. Structural parameters of flexible photonic crystals can be readily modified.

## 3. Plasmonic Assisted Full-Color Devices

Plasmon-assisted nanophotonic devices have drawn particular attention in recent years because of their tremendous latent capacity for new imaging and display technologies [86,87,88] since these devices hold great promise for furnishing the breakthroughs which are necessary for the next-generation photonics technology. Manipulating surface plasmons in visible frequencies is critical and essential. Active plasmonic color filters [89] have also been demonstrated experimentally using liquid crystals (LCs) since it is easy to obtain a noticeable refractive index change. In this section, we summarize and categorize the practical achievements of plasmon-based/assisted color filtering devices.

### 3.1. Transmissive Plasmonic Color Filters

Using simple nanostructures, various color filters have been demonstrated, including nanoslit antennae [3], ultrathin gratings [7], coaxial apertures [28], and metal-insulator-metal [5,90] resonators. Different colors could be separated after passing through plasmonic apertures from a broadband light source because plasmon resonance is highly sensitive to geometric parameters. One typical design is the coaxial annular aperture fabricated in noble metals (gold and silver, for instance). Furthermore, coaxial structures can form single layer metamaterials and support propagating plasmon modes in a wide range of wavelength bands. In the transmission spectrum of coaxial apertures, normally two kinds of resonant peaks are revealed and both of them can be finely tuned by controlling coaxial apertures. They are cylindrical surface plasmons (CSPs) and planar surface plasmons (PSPs). CSPs are dependent on structural design and metal thickness while PSPs are sensitive to the periodicity of the ring array. The coaxial apertures act as Fabry-Pérot resonators, resulting in resonant peaks in the transmission spectra. By adjusting the aperture width (gap between the outer and inner radii), one could simply shift the cut-off frequency of guided modes.

**Figure 4:**
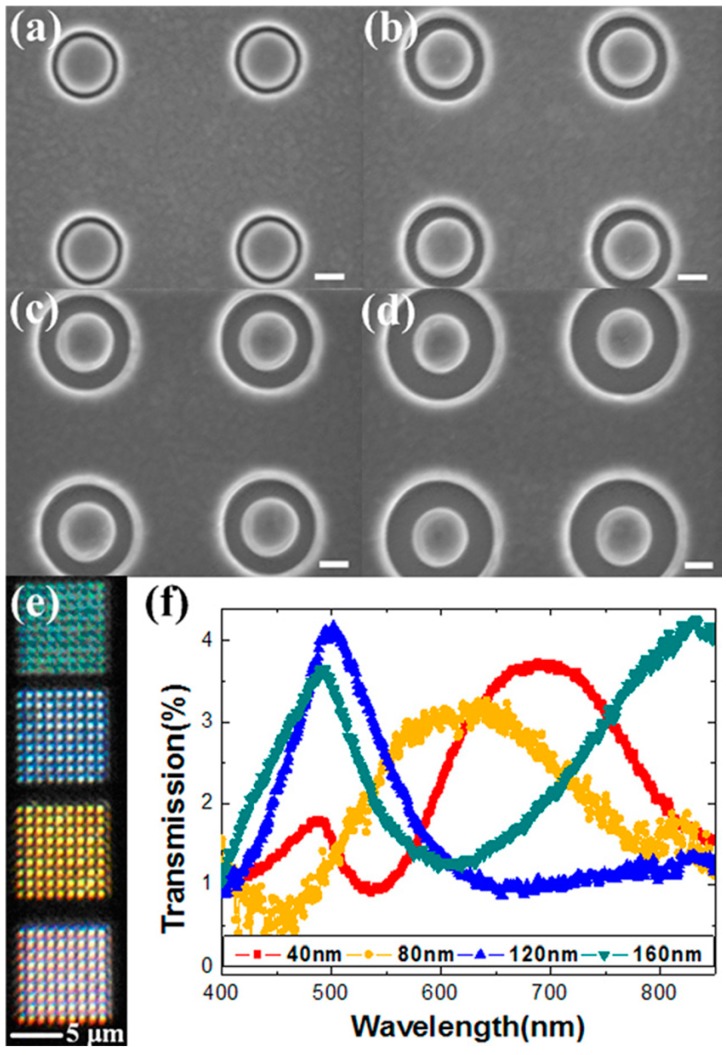
(**a**–**d**) SEM images of the fabricated coaxial apertures with (**a**) 40 nm; (**b**) 80 nm; (**c**) 120 nm; and (**d**) 160 nm gap width (outer radii equal to 240 nm, 280 nm, 320 nm, and 360 nm with 200 nm fixed inner radius). Note that the pitch is fixed at 1200 nm for (**a**–**d**). Scale bars, 200 nm. (**e**) Corresponding optical image presenting different colors; (**f**) Measured transmission spectra of the coaxial apertures with different color outputs. All figures are adapted from reference [28].

As shown in Figure 4, coaxial apertures with varying gap widths (40 nm, 80 nm, 120 nm, and 160 nm) were fabricated by focused ion beam (FIB) patterning in a 160 nm thick gold film supported by a quartz substrate. Under broadband white light source illumination, individual colors can be filtered out as illustrated in Figure 4e. Using a microspectrometer, the corresponding transmission spectra were obtained by normalizing the intensity to light through a bare quartz substrate, as plotted in Figure 4f. One can see that the intensity is relatively low (less than 5%) due to a thick metal film (160 nm gold) in this case. The full width at half maximum of the transmission peak becomes broader with increasing wavelengths. Note that the filtering efficiency can be significantly improved by optimizing the geometrical parameters of the device, e.g., film thickness, materials, inner and outer radii of the coaxial apertures. Poujet and coworkers showed that over 90% intensity could be achieved at a certain frequency by using silver [91].

### 3.2. Reflective Plasmonic Color Filters

**Figure 5:**
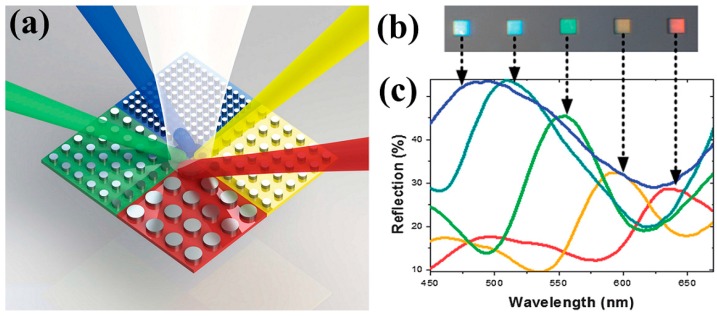
(**a**) Schematic drawing of silver nanorod color filters working under reflection mode; (**b**) optical image showing the reflective colors from different silver nanorod arrays; (**c**) measured reflection spectra of the corresponding arrays as a function of wavelength. All figures are adapted from reference [25].

The development of plasmon related/enhanced reflective filtering devices is mainly limited by efficiency since metals have significant absorption at visible frequencies. To receive acceptable reflected energy, normally, high aspect ratio structures (large height and small gaps) are needed, resulting in challenging fabrication processes. Normal lift-off processes are only effective for thin devices because resists can be hardly removed if they are covered by thick metal films. One feasible solution to overcome the difficulties mentioned above is to use a dry etching method to transfer patterns (top-down fabrication process). By using argon ion milling, large aspect ratio silver nanorods were fabricated [25] and reflective color filters could then be experimentally demonstrated, as presented in Figure 5. The working mechanism is simple. The plasmon resonance in the reflection spectra shifts to shorter wavelengths with decreasing array periodicities, as illustrated in Figure 5a.

To experimentally demonstrate the reflection-mode color filtering devices, high aspect ratio nanorod arrays were fabricated in silver films by using EBL followed by argon ion milling. Inter-rod spacing of 20 nm smallest was achieved to reveal blue color as shown in Figure 5b with 320 nm array periodicity (300 nm rod diameter), leading to more than 50% reflected intensity as can be seen in Figure 5c. Interestingly, the full width at half maximum for reflection peaks becomes broader with larger wavelength while the intensity decreases from more than 50% to around 32% (yellow) and 26% (red), respectively. This is due to a non-uniform surface (rough and oxidation of metal film). Moreover, oblique sidewalls may also affect the optical properties dramatically because non-vertical cross-sections may influence scattering and absorbance significantly. However, straight sidewalls are almost impossible due to redeposition effects. Based on the inter-rod separations, there are two types of plasmon resonances generated. Namely, they are weak coupling and strong coupling regimes. For relatively large separations, weak coupling between neighboring nanorods occurs and typically small reflected energy can be generated. On the other hand, strong coupling reveals ultra-small inter-rod separations and the reflection can be enhanced remarkably, leading to high efficiency color filtering devices. Note that in the weak coupling regime, the geometry of the nanorod is the most important parameter. In the strong coupling regime, however, both the inter-rod distance and the ratio between pitch and wavelength dominate the optical response. In addition, all the geometrical parameters of the nanorod arrays are highly adjustable thanks nowadays to the mature nanofabrication technologies, resulting in highly flexible optical platforms which can be easily integrated into modern display and imaging techniques for nanophotonics and integrated optics.

### 3.3. Angle-Dependent Color Filters

Structural color pixels can outperform their chemical counterparts since they can bear long-time constant illumination with stronger light intensities. In addition, they can achieve high compactness and resolution beyond the diffraction limit with wide tunability and highly stable device performance. Moreover, structure color filtering devices can enable high efficiency and low power consumption with dramatically slimmed dimensions and enhanced resolution. However, the incident angle tolerance limits the practical applications of such optical components. To achieve more promising applications, thinner spectral response is desired for vivid colors with high angle tolerance. Researchers have made every effort to realize angle robust optical filters with narrow broad spectral response and high optical performance to yield a superior color contrast. To obtain angle-insensitivity, one has to avoid relying on grating coupling for plasmonic mode excitation. In contrast to grating coupling, plasmon assisted resonators and antennae have been experimentally demonstrated as candidates for structure colors which have advantageous features such as brilliancy and color vividness. Gratings in metal surfaces without involving diffraction effects can be used to control the light polarization so that it is possible to use an analyzing polarizer to manipulate the transmitted colors, which are largely independent of the angle of incidence. Significant work remains to develop a reflective display that can provide a bright and full-color image comparable to printed media with low power consumption as a new class of display element, which allows the full-color emission from a single pixel under a broad range of ambient lighting conditions. However, one can still obtain individual colors based on tuning the incident angle of white light illuminated onto nanostructures. Instead of adjusting geometry parameters, different color outputs can be achieved via incidence scanning with a fixed design. Using a single plasmonic chip, photon-plasmon coupling interactions can be triggered and further engineered to reach continuous color tuning effects across the whole visible range [92].

**Figure 6:**
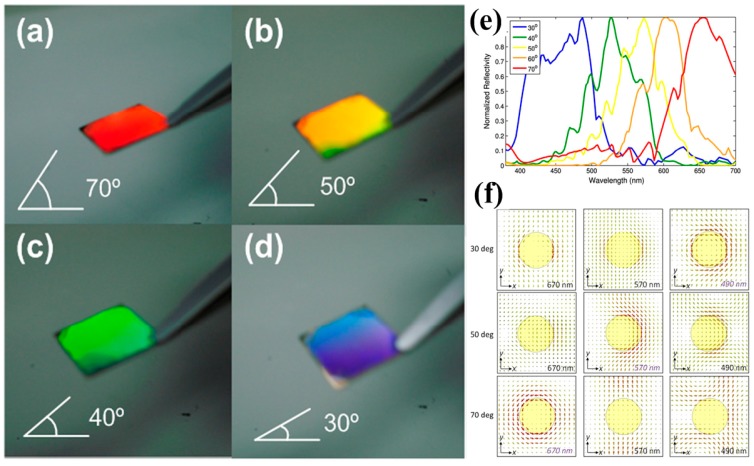
Optical images of the same sample of gold nanorods on a silicon substrate taken from varying angles. Different vivid colors of (**a**) red, (**b**) yellow, (**c**) green, and (**d**) blue are shown. Redshift of resonance colors with increasing tilting angles can be clearly observed. (**e**) Normalized specular reflections for various angles of incidence. (**f**) Magnetic field distributions near the interface between the nanorod array and the silicon substrate at varying incident angles of 30 (top row), 50 (middle row), and 70 (bottom row) degrees and different wavelengths of 670 (left column), 570 (middle column), and 490 (right column). All figures are adapted from reference [92].

A large functional area (1 × 1 cm^2^) can be realized by using interference lithography to define patterns and ion milling to transfer patterns. As demonstrated in Figure 6a–d, red, yellow, green, and blue colors can be filtered out at varying angles. Resonant peaks can blue shift with decreasing angles and this can be verified by calculations as plotted (normalized to the maximum reflectivity at each respective incident angle) in Figure 6e. Figure 6f presents the magnetic field distributions at 30° (top row), 50° (middle row), and 70° (bottom row). One can see that a curl-like pattern is revealed at the plasmon resonance wavelength for different incident angles.

**Figure 7:**
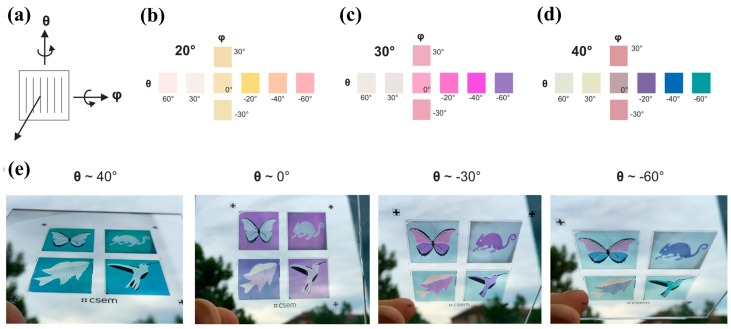
(**a**) Sketch illustration showing sample orientation and two rotating directions (θ and φ); (**b**–**d**) Measured colors of the samples for different evaporation angles and different viewing angles θ and φ defined in sketch (**a**); Note that colors mainly appear at negative angles θ. (**e**) Glass substrates with 4 different sample areas (2 cm × 2 cm) created by the evaporation angles 20°, 30°, and 40°. Note that images were taken in front of a cloudy sky and with unpolarized light. All figures are adapted from reference [93].

Using tilted aluminum nanowires with broken symmetry, Duempelmann and co-workers [93] showed that a color rendering substrate can be materialized in one tilt direction due to angle-dependence of plasmon resonance interactions with propagating modes. By fine-tuning the fabrication steps, high throughput and large scale color rendering substrates as shown in Figure 7 were obtained at relatively low cost due to symmetry breaking. Note that the color tuning effect can be realized with different viewing angles θ and φ. However, the color change is weak when rotating the substrate vertically (varying φ, tilt along the periodic nanoarrays) due to the orientation of nanowires.

### 3.4. Depth Variant Color Filters

**Figure 8:**
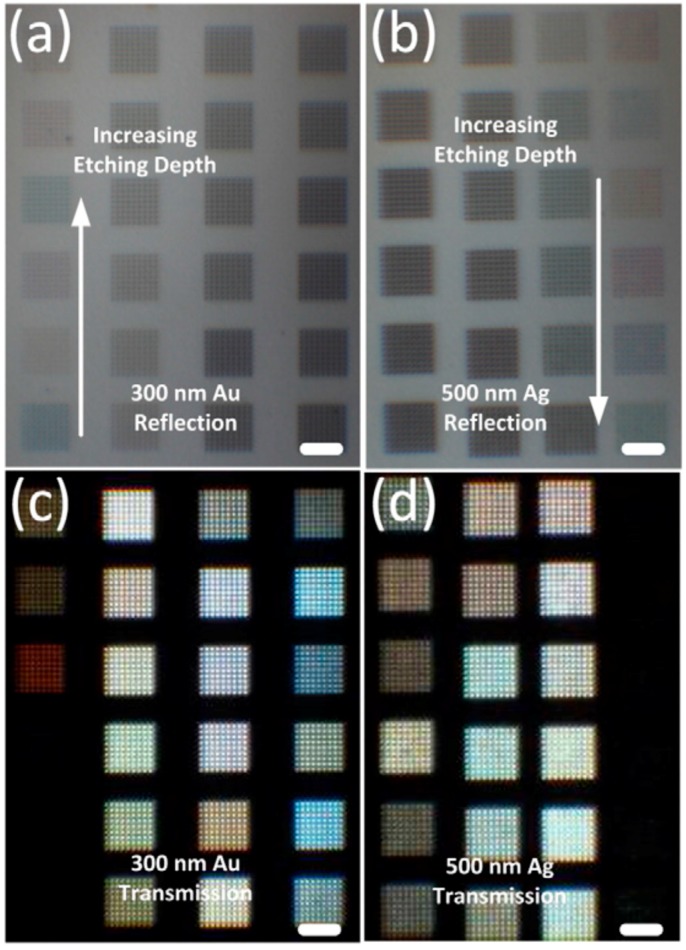
Optical images for (**a**) 300 nm gold under reflection mode; (**b**) 500 nm silver under reflection mode; (**c**) 300 nm gold under transmission mode, and (**d**) 500 nm silver under transmission mode with varying etching depths of coaxial apertures. Scale bars, 5 μm. All figures are adapted from reference [94].

Coaxial nanocavities which can support propagating plasmons are capable of filtering individual colors with precise adjustment of etching depth [94]. Varying surfaces can present changing color outputs at certain frequency bands under both transmission and reflection modes. FIB lithography has been used to accurately manipulate the etching depth. Note that bumped surfaces will lead to blurry images because of inevitable redeposition effects. It is also worth mentioning that the colors revealed are not as vivid as above using other working mechanisms since the plasmon tuning effect in both reflection and transmission is relatively weak and within a very small range of wavelength band. However, it works for both gold and silver films with different thicknesses (300 nm and 500 nm in this case). Note that thinner spectral response is desired for generating vivid colors due to the high purity of the monochrome. Researchers have made every effort to narrow the broad spectral response to achieve devices with high optical performance. From Figure 8, one can see that the color turns to gray/black when apertures are drilled through the film. This concept of intaglio metamaterials/metasurfaces enables 2D platforms which can manipulate light in a confined plane without breaking the surface integrity. Another remarkable advantage is that hollowed apertures are suitable for large-scale production using interference or other lithography techniques.

### 3.5. Dynamically Tunable Plasmonic Color Filters

**Figure 9:**
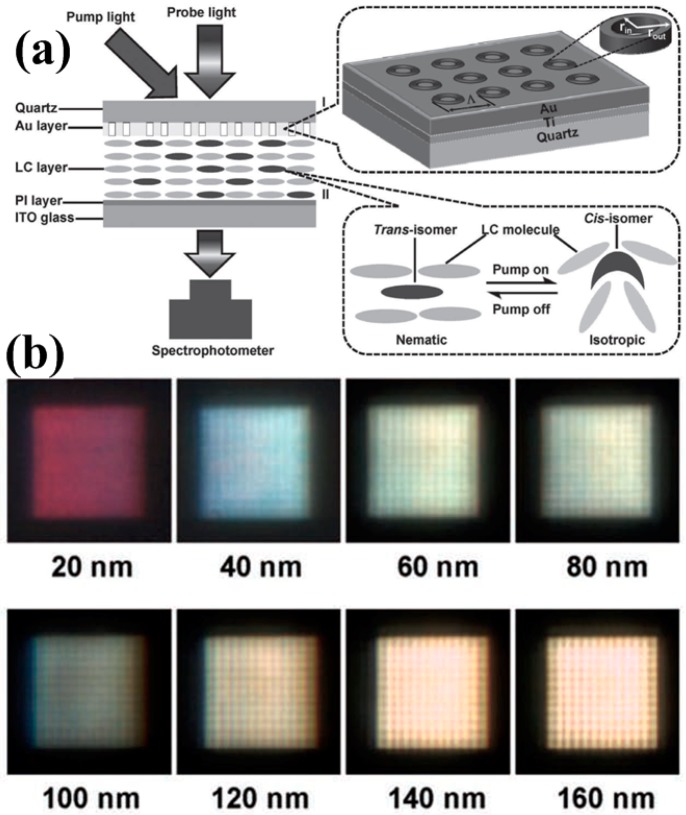
(**a**) Schematic of the tunable coaxial color filter structure and experimental setup using liquid crystals (LCs); (**b**) charge-coupled device (CCD) captured colors with varying aperture sizes showing different outputs. All figures are adapted from reference [89].

Actively tunable devices are always desired for dynamic adjustment and versatile control. LC is a perfect candidate [95,96,97,98,99,100,101,102,103,104,105,106,107,108] for tunable devices because of its unique optical properties and low cost. Using LC-based working mechanisms, plasmonic color filters can be easily combined with most of the existing display and imaging technologies or even integrated into commercially available optical components/platforms. Coupling between photons and plasmons can be precisely engineered by manipulating the LC properties via an external electric field or thermal control. Different phase status is switchable to further control the color outputs [89]. As shown in Figure 9a, one can see that the gold coaxial apertures supported by a quartz substrate are covered with an LC layer which can shift status between nematic and isotropic with the pump light (flood exposure using a UV light source) on/off. The photoisomerization of LC molecules is completely reversible and reproducible with either visible light irradiation or thermal isomerization (cis-isomer can be converted to the trans-isomer form). The interaction between the alignment of LC molecules and color filter geometry plays a crucial role in determining the LC properties (effective refractive index, for instance) and therefore further affects the subsequent peak shift and enhancement in transmission spectra, as illustrated in Figure 9b.

**Figure 10:**
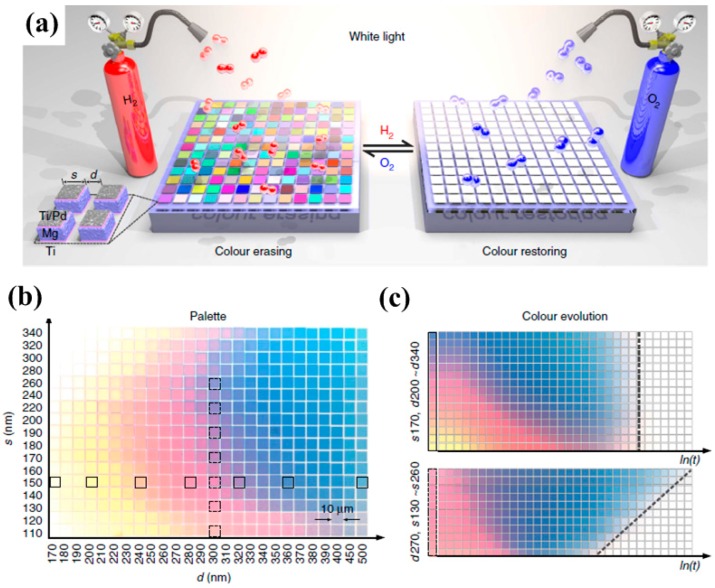
(**a**) Schematic view showing the working principle of the plasmonic metasurface composed of hydrogen-responsive magnesium metasurfaces interacting with incident unpolarized white light; (**b**) color palette obtained by stepwise tuning of s and d; (**c**) color evolutions of the selected color squares upon hydrogen exposure over time *ln*(*t*). The grey-dashed lines indicate the color vanishing times in the two cases. All figures are adapted from reference [109].

Using catalytic magnesium metasurfaces, Duan et al. [109] demonstrated dynamic color pixels which can be switched on/off by exposing to hydrogen or oxygen. Hydrogenation/dehydrogenation of magnesium metasurfaces will happen subsequently, enabling invertible color restoring and erasing processes as illustrated in Figure 10. More importantly, this dynamic animation concept is perfect for colorimetric sensing, high-security information encryption, and anti-counterfeiting applications with sub-wavelength resolutions.

## 4. Conclusions and Outlook

We summarized the recent development of photonic crystal based and plasmon-assisted color filtering devices and their potential applications. Pixels with ultrasmall sizes are promising for sub-wavelength resolution display and imaging techniques. Thanks to the fast development of nanofabrication technologies, high density (pixel per inch, PPI) devices are enabled which may pave the way for next generation display productions. However, researchers still need to further improve the optical performance of color filtering devices for more practical applications. Filters with both high transmission intensity and narrow passing band are critical to produce high throughput and low-cost optical components for new optical devices. Additionally, high speed actively tunable devices are extremely important for future development of multiple-functional assemblies. In practice, low-cost and high-throughput approaches are desired because they are more suitable for industrial applications.
